# Multidisciplinary integration in the context of integrated care - results from the North West London Integrated Care Pilot

**DOI:** 10.5334/ijic.1146

**Published:** 2013-10-23

**Authors:** Matthew Harris, Felix Greaves, Laura Gunn, Sue Patterson, Geva Greenfield, Josip Car, Azeem Majeed, Yannis Pappas

**Affiliations:** Public Health, Department of Primary Care and Public Health, Imperial College London, 3rd Floor, Reynolds Building, St Dunstan's Road, Hammersmith, London W6 8RP, UK; Department of Primary Care and Public Health, Imperial College London, 3rd Floor, Reynolds Building, St Dunstan's Road, Hammersmith, London W6 8RP, UK; Biostatistics & Department Chair of Integrative Health Science, Stetson University, 421 North Woodland Blvd, 149A Sage Hall, DeLand, FL 32723, USA; Metro North Mental Health, University of Queensland, Brisbane, QLD 4072, Australia; Department of Primary Care and Public Health, Imperial College London, 3rd Floor, Reynolds Building, St Dunstan's Road, Hammersmith, London W6 8RP, UK; Department of Primary Care and Public Health, Imperial College London, 3rd Floor, Reynolds Building, St Dunstan's Road, Hammersmith, London W6 8RP, UK; Primary Care, Department of Primary Care and Public Health, Imperial College London, 3rd Floor, Reynolds Building, St Dunstan's Road, Hammersmith, London W6 8RP, UK; PhD School, Institute for Health Research, University of Bedfordshire, University Square, Luton, Bedfordshire LU1 3JU, UK

**Keywords:** health care delivery, communication, health services, multidisciplinary groups

## Abstract

**Background:**

In the context of integrated care, Multidisciplinary Group meetings involve participants from diverse professional groups and organisations and are potential vehicles to advance efficiency improvements within the local health economy. We advance a novel method to characterise the communication within Multidisciplinary Group meetings measuring the extent to which participants integrate and whether this integration leads to improved working.

**Methods:**

We purposively selected four Multidisciplinary Group meetings and conducted a content analysis of audio-recorded and transcribed Case Discussions. Two coders independently coded utterances according to their ‘integrative intensity’ which was defined against three a-priori independent domains - the Level (i.e. Individual, Collective and Systems); the Valence (Problem, Information and Solution); the Focus (Concrete and Abstract). Inter- and intra-rater reliability was tested with Kappa scores on one randomly selected Case Discussion. Standardised weighted mean integration scores were calculated for Case Discussions across utterance deciles, indicating how integrative intensity changed during the conversations.

**Results:**

Twenty-three Case Discussions in four different Multidisciplinary Groups were transcribed and coded. Inter- and intra-rater reliability was good as shown by the Prevalence and Bias-Adjusted Kappa Scores for one randomly selected Case Discussion. There were differences in the proportion of utterances per participant type (Consultant 14.6%; presenting general practitioner 38.75%; Chair 7.8%; non-presenting general practitioner 2.25%; Allied Health Professional 4.8%). Utterances were predominantly coded at low levels of integrative intensity; however, there was a gradual increase (*R* ^2^ = 0.71) in integrative intensity during the Case Discussions. Based on the analysis of the minutes and action points arising from the Case Discussions, this improved integration did not translate into actions moving forward.

**Interpretation:**

We characterise the Multidisciplinary Groups as having consultative characteristics with some trend towards collaboration, but that best resemble Community-Based Ward Rounds. Although integration scores do increase from the beginning to the end of the Case Discussions, this does not tend to translate into actions for the groups to take forward. The role of the Chair and the improved participation of non-presenting general practitioners and Allied Health Professionals seems important, particularly as the latter contribute well to higher integrative scores. Traditional communication patterns of medical dominance seem to be being perpetuated in the Multidisciplinary Groups. This suggests that more could be done to sensitise participants to the value of full participation from all the members of the group. The method we have developed could be used for ongoing and future evaluations of integrated care projects.

## Introduction

Integrated care brings health professionals together to improve outcomes for patients and carers [1,2] and is considered particularly important for patients with multiple chronic diseases, who may need care from a range of services or departments. Cross-agency integration [3], also known as virtual integration [1], is often achieved through the use of Multidisciplinary Groups and involves participants who are not only from different professional groups but also from different organisational backgrounds. In a typical Multidisciplinary Group, participants discuss not only complex care of individual patients, but also have the potential to discuss the ways to improve working within that local health economy, i.e. working in a more integrative manner. However, this potential may not be realised for various, often complicated reasons and there might be a personal or professional preference to restrict discussion to the care of the patient being presented rather than engaged with the thorny issues of organisational integration. Whilst it may be necessary, it is not sufficient for professionals to meet and discuss cases for integration to occur. The degree to which they achieve integrated working is likely to be dependent on membership, structure, leadership and capacity to develop shared values.

Effective communication is therefore key to cultivating an integrative way of working. It allows a Multidisciplinary Group participant to transcend their inclination towards their own field and find common inter-professional territory [4]. It follows that communication patterns may also be useful in describing how the Multidisciplinary Groups are working, yet we have found no example of empirical research that measures the degree to which participants are integrating. In this study, we advance our previously developed methodology [10] and explore the communication patterns within Multidisciplinary Groups of a large-scale Integrated Care Pilot, investigating the degree to which Case Discussions within Multidisciplinary Groups foster an integrative way of working between the participants.

## Materials and methods

### Context

The North West London Integrated Care Pilot, launched in June 2011, brings together over 100 general practices, two Acute Trusts, five Primary Care Trusts, two Mental Health Trusts, three Community Health Trusts, five local authorities and two voluntary sector organisations to improve the coordination of care for a pilot population of 550,000 people. Specifically, the Integrated Care Pilot serves people aged over 75 years and those with diabetes. Although still in the early stages of implementation, the Integrated Care Pilot has already received national awards for its innovations in design and delivery [5] and been the subject of intense media interest [6,7].

As described in detail elsewhere [8], the Integrated Care Pilot was established to improve care coordination across several service levels and organisations and develop pro-active case management of complex patients identified to be at high risk of hospitalisation. Multi-Disciplinary Group meetings are a core component of the Integrated Care Pilot. Sixteen Multidisciplinary Groups meet around once per month in 10 different localities. Participants of each Multidisciplinary Group include general practitioners, Acute Care Consultants and Allied Health Professionals (District Nurses, Community Matrons and Social Workers) from different Acute Trusts, Community Care organisations and general practices within the local health economy - an attempt to deliver virtual (i.e. not involving actual mergers), vertical and horizontal integration [1,9]. The primary purpose of these groups is to, each month, discuss the care of a selected group of complex patients and elaborate carefully considered care plans that will improve the care and its coordination for these patients and ultimately to reduce hospitalisations. In a typical Multidisciplinary Group meeting, participating general practitioners will take it in turns to each present one of their complex patients for discussion by the group.

Secondary, but still important, objectives are for the Multidisciplinary Groups to serve as a forum for all participants to learn more about the local health services, their remits and functions. In doing so, participants can explore methods for improving collaborative working and improve health care services and their coordination generally, not only just for the selected complex patient subject of the Case Discussion, but also for the local health economy as a whole. Early on in the Integrated Care Pilot, this objective was clearly articulated:

the MDGs are a *vehicle* for delivering productivity and efficiency improvements within and across the various providers…[participants should] move away from stereotypes, get to know each other, be reflective and responsive, increase the level of trust, coordination and collaboration across providers working together towards better patient care… (NWL ICP presentation August 2011 [authors’ emphasis added])

### Conceptual framework

As described elsewhere [10], we defined ‘integrating’ as the process within the Multidisciplinary Group meeting that enables or promotes an improved collaboration, improved understanding, and improved awareness of self and others within the local health care economy such that efficiency improvements could be identified and action could be taken. The literature on effective team-working, decision-making, communication and inter-professional care provides some insight into the group dynamics that would support such a process. Bales’ Interaction Process Analysis [12] is one of the most widely applied measures of group decision-making and enables the assessment of participants’ interaction style in terms of whether it is positive, constructive and supportive, or whether there is antipathy and tension. Bales’ model is grounded in the view that utterances that are solution-oriented, supportive, offering opinions and exhibiting empathy are much more likely to improve the dynamics between the participants [11–14]. Hence, they are an important first step towards integrated care. We draw also on Clark (1997) [15] who notes that ‘effective inter-professional working in multi-disciplinary teams requires individuals to be reflexive in their communication’. This enables participants to transcend their own professional roles and routines, leading to learning and a more collaborative environment, also an important step towards integration [15]. Finally, Curry and Ham [1] note that health service integration can occur, on various levels - micro (the individual patient level), meso (groups and services) and macro (organisations). Professionals, services and organisations may work in an integrated way around the care of an individual patient, but this may not extend to other patients, or to general structures and processes.

We conceptualise ‘integrating’ communication patterns as a product of these three domains or axes.

- the *type* of interaction between the participants (we call this the Valence and it has three subtypes: Problem, Information and Solution).
- the degree of *reflexivity* that participants are exhibiting (we call this the Focus and it has two subtypes called Concrete and Abstract).
- and the *content* of the conversations (we call this the Level and it has three subtypes called Individual, Collective and Systems).

### Methods

We applied the methods described in detail in Harris et al. (2012b) [10]. From all the aspects of the studies undertaken within the North West London Integrated Care Pilot Evaluation, appropriate ethical and governance approvals were sought from the National Health Service, National Research Ethics Service for City and East London. In accordance with the harmonised Governance Arrangements for Research Ethics Committees which came into effect in September 2011, the studies were granted ethics approval (ref. 11/LO/1918). After consultation with local Research and Development offices at Imperial Academic Health Science Centre, exemption for local Research and Development approvals was granted on the grounds that the activities within the studies (Multidisciplinary Group observations, for example) constituted part of a service evaluation, not involving patients and did not take place on an National Health Service site. Consent to support and take part in activities within the North West London Integrated Care Pilot contributing to its evaluation was part of the agreement in organisations being involved in the Pilot. Although consent was implicit in being invited to undertake the evaluation at Multidisciplinary Group meetings, in order to ensure we met the best practice standards, we sought and obtained verbal consent from the individual professionals that were present; however, there was no specific ethical requirement to do so, nor to obtain written consent. We selected purposively four Multidisciplinary Groups in different sites each with a different Multidisciplinary Group coordinator. In accordance with the ethics protocol, prior to Multidisciplinary Group observations, we sought written approval for observation from Multidisciplinary Group chairs, and provided participant information sheets to all participants in advance of the observations. We allowed time to answer questions and concerns in advance of and at the time of the meetings, and made explicit that where consent is withheld by any single individual then the meeting observation would not proceed. No inducements were provided, and consent was obtained unanimously and verbally at the time of the meeting. We audio-recorded one Multidisciplinary Group meeting in each site.

One participant (M.H.) was a non-participant observer during each meeting, however had some interaction with participants prior to the meeting. Ethical approval for the research was obtained from the National Health Service Research Ethics Committee as part of a larger mixed methods evaluation of the North West London Integrated Care Pilot [16]. Full-verbatim transcripts were obtained within a week of each meeting from a certified medical transcribing company under strict confidentiality. The transcripts were quality assured within one week of receipt to confirm that each quote was attributed to the correct individual. The transcripts were then anonymised in place and person and any patient-identifiable information within the transcript was removed or anonymised. The transcripts then underwent a ‘treatment’ to separate discussion and dialogue into utterances or units of meaning. An utterance is defined as a phrase or sentence expressing a complete thought, identified linguistically based on intonation [17]. Dialogue was divided into sentences or phrases of approximately equal length or where significant shifts in meaning, object or subject occurred within the dialogue as illustrated by the example below (Multidisciplinary Group 2, general practitioner 6):

He has had physiotherapy at St Mary's Hospital./

From there they have referred to Westminster Rehabilitation Centre…/

but at times he's adamant he does not follow the physiotherapists, occupational therapists./

He has got a daughter who's very much concerned./

He has got recurring falls.

A second researcher (F.G.) checked where the units of meaning began and ended and any disagreements were resolved through discussion and consensus. The two researchers (F.G. and M.H.) independently coded the transcripts. All utterances in a transcribed Case Discussion were given a trivalent Event Code, by coding first with respect to the Level, then with respect to the Valence and finally Focus so that any bias to code preferentially towards one permutation of the three codes was minimised. Partial or incomplete interjections, mumblings or verbiage, e.g. ‘Pardon?’, ‘Yes’, ‘No’, ‘Mmm’ and ‘Sorry?’ were given a code 0.

Once the transcripts had been divided into units of meaning, they were coded according to the speaker type (i.e. presenting general practitioner, non-presenting general practitioner, Chair, Consultant, District Nurse, Community Matron, Social Worker, Multidisciplinary Group coordinator) and the integrative intensity of the utterance [10]. We calculated the standardised weighted mean integrative intensity score in each decile of the Case Discussions to ascertain whether participants were ‘integrating’ over the course of the discussion, i.e. we measured whether Case Discussions that begin, understandably, with a case presentation and discussion of individual issues pertinent to the care of that patient, progressed to ‘higher’ levels of abstraction, reflection and interaction, discussing issues shared and common to similar cases and furthermore to issues shared and common to all participants and their organisational domains.

### Inter-coder and intra-coder reliability

The coding scheme was tested for inter- and intra-rater validity using Kappa scores (Table 1). We used a randomly selected Case Discussion to ascertain our Kappa scores between the two coders (inter-rater), and also between two repeated coding of the same Case Discussion by the same coders (intra-rater) performed several weeks apart. Sim and Wright (2005) [18] have shown that chance agreement is affected by the number and prevalence of the codes and that Kappa scores should be adjusted for prevalence and interpreted in the context of the maximum Kappa obtainable. We therefore calculated a Prevalence and Bias-Adjusted Kappa score [19] to ascertain the relative importance of both and their impact on the Kappa. We also calculated a Maximum Kappa for comparison so that we had a reference value for Kappa against which the Kappa and Prevalence and Bias-Adjusted Kappa score could be compared. All of our codes were independent, avoiding a potential Kappa inflation. For pragmatic reasons, and given the good agreement ascertained by the Kappa scores, the Case Discussions were divided equally between the two coders.

## Results

**Figure 1.**
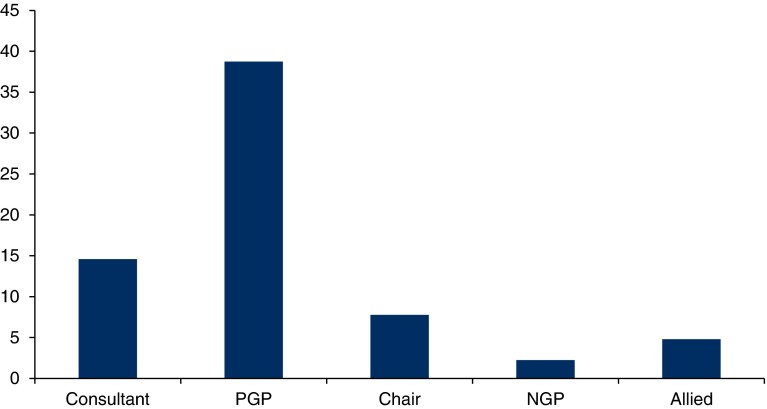
Distribution of utterances (%) per participant type and MDG (For all MDGs Consultants = 2, PGP = 1, Chair = 1; For MDG 1 NGP = 6, MDG 2 NGP = 4, MDG 3 NGP = 3 and MDG 4 NGP = 4; For MDG 1 Allied = 3, MDG 2 Allied = 3, MDG 3 Allied = 5, MDG 4 Allied = 2). MDG, Multidisciplinary Group; NGP, non-presenting general practitioner.

The four observed Multidisciplinary Groups (one Diabetes and three Elderly care) meetings encompassed 23 Case Discussions and over seven hours of discussion. The number of people attending the Multidisciplinary Group meetings ranged from 11 to 15 (mean 14). Recordings yielded 4209 utterances, with a mean of 183 (Standard deviation 98.8) utterances per Case Discussion. Each utterance was coded three times - Level, Valence and Focus - corresponding to around 400 pages of verbatim discussion. Only 6.9% of all utterances could not be coded and this did not vary significantly by Case Discussion or Multidisciplinary Group. Because numbers of general practitioners and Allied Health Professionals present in Multidisciplinary Groups varied, we calculated the utterance rate per participant type to enable comparison (Figure 1). In Multidisciplinary Group 4, the Multidisciplinary Group coordinator acted as the Chair as the usual general practitioner was not present. As less than 0.5% of all the utterances were made by the Multidisciplinary Group coordinators in the other Multidisciplinary Groups, we excluded these from the analysis. Case Discussions were all dominated by the Consultants and the presenting general practitioners, with little involvement from general practitioners not presenting the case or other attendees. There was no significant difference in this pattern across the four Multidisciplinary Groups.

**Figure 2.**
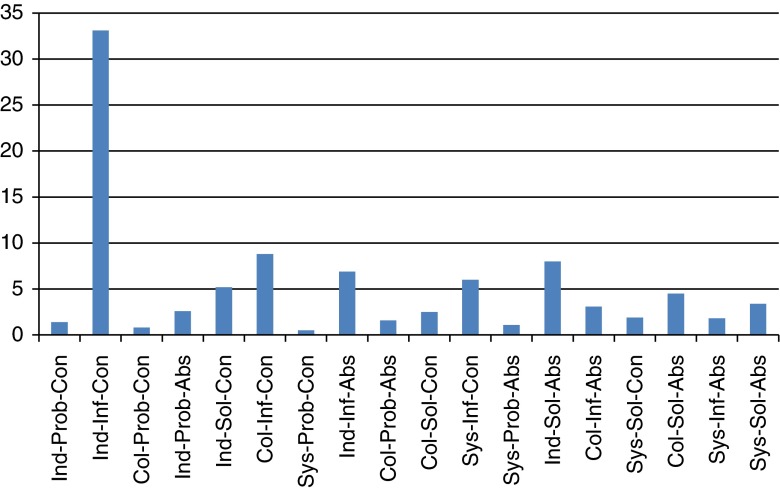
Proportion of all utterances (all MDGs) by trivalent Event Code domain (does not include uncodeable utterances). Ind, Individual; Col, Collective; Sys, System; Prob, Problem; Inf, Information; Sol, Solution; Con, Concrete; Abs, Abstract.

Figure 2 shows the distribution of utterances according to their trivalent Event Code across all the Multidisciplinary Groups. The integrative intensity of Multidisciplinary Groups was generally low. Although some time was spent on higher levels of abstraction at the collective and systems levels, emphasis was on the exchange of patient- or individual-level information and orientation (Ind-Inf-Con). Overall, although the talk was commonly solution - rather than problem-oriented, the individual focus reduced integrative intensity for the group as a whole. This pattern was consistent across Multidisciplinary Groups.

**Figure 3.**
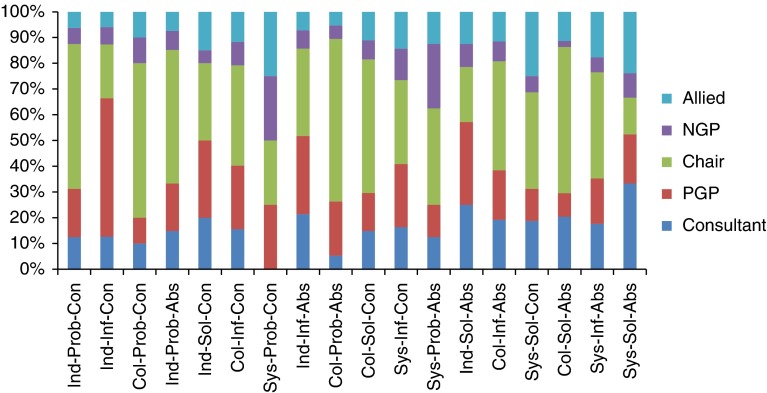
Proportion of Event Codes by participant type for all MDGs (does not include uncodeable utterances). Ind, Individual; Col, Collective; Sys, System; Prob, Problem; Inf, Information; Sol, Solution; Con, Concrete; Abs, Abstract.

The emphasis on the lower integrative potential Event Codes was driven, largely, by the Presenting general practitioner (Figure 3) demonstrating that the case presentation comprises a large proportion of the talk. Allied Health Professionals contribute substantially less to the discussion than the other participant types; however, their utterances are predominately more integrative - accounting disproportionately for higher Event Codes, in particular Sys-Sol-Abs.

**Figure 4.**
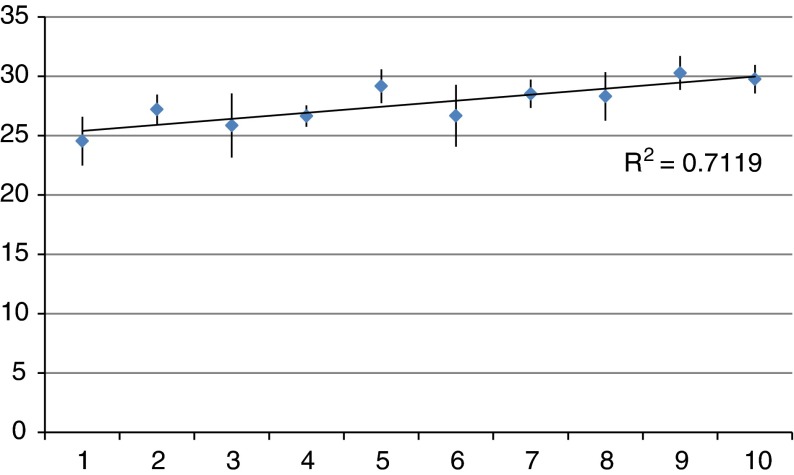
Average Standardised Weighted Integration Intensity Score per time decile of Case Discussions (with standard deviation shown).

Finally, Figure 4 shows the distribution of the utterances with respect to their integrative intensity throughout Case Discussions. Here, we have used the standardised weighted means of integration intensity for each decile, across all Multidisciplinary Groups, using the mean weighting scale described in Harris et al. (2012) [10]. In each Multidisciplinary Group, the integrative intensity of each Case Discussion exhibited broadly similar characteristics. They began at low levels of integrative intensity, a function of the case presentation, but the integrative intensity increased overall by the end of the discussion. As a result of presenting an individual case, participants began to consider broader, collective or systems implications, or reflectively considered possible solutions to service constraints within the local health economy. Although Case Discussions fostered this apparent level of ‘integrating’, only two of the several dozen action points identified during all Case Discussions were ‘beyond’ the care of the individual case - to distribute a directory of services and to send some information on community care alarms to the Multidisciplinary Group participants.

## Discussion

Beyond improving the care of individuals, the objective of the Multidisciplinary Groups was to foster a ‘miasmic’ cultural change and enhance integration more broadly. This is important because in a forum such as a Multidisciplinary Group in the integrated care context, Case Discussions are potential opportunities for participants to examine the broader inefficiencies and challenges in the inter-organisational environment. From the Case Discussions analysed in this study, we found that talk was predominantly at low Event Codes. Although there was evidence that participants were, overall, integrating as a result of the Case Discussions, i.e. during a discussion, there was a gradual shift from low integrative intensity, focusing mainly on the presentation of the complex case, to higher integrative intensities, concerning collectives of patients and discussion of issues within the local health economy, this did not translate into plans of action and activity typically remained focused on individual patients.

Participation in Case Discussions was restricted primarily to Consultants and the presenting general practitioners. Although there are more Allied Health Professionals in each group, they each tended to contribute substantially less to the discussion. As they accounted for a large proportion of utterances coded at a higher integrative level, enhancing their participation could contribute to achievement of virtual integration. Also noteworthy is that general practitioners, other than the one presenting the case, contribute little to discussion. Based on our analysis, we characterise the Integrated Care Pilot Multidisciplinary Groups as having consultative characteristics with some trend towards collaboration [20], but can best be described as resembling Community-Based Ward Rounds. The traditional communication patterns of medical dominance permeate and are being perpetuated in the Multidisciplinary Groups, precluding the opportunity to work more inclusively, collectively and in a truly multi-disciplinary way. This suggests that more could be done to sensitise participants to the value of full participation from all members of the group, and for the Chairperson and Multidisciplinary Group coordinators to capture opportunities to explore learning from individual cases to other similar cases and how services could work better together to improve the care generally. This could range from the identification of simply a new pro-forma to, at the other end of the spectrum, a new service - all working towards an improved and more integrated way of working between autonomous organisations.

Improvements in patient care more broadly require a very different kind of communication - one based on collaboration and integration [21]. The ability and aspiration for health professionals to contribute to the conversation are key to bringing broader health system issues to the fore. There are many obstacles to developing the kind of communication needed for effective inter-professional working in a Multidisciplinary Group - these include overcoming medical dominance [22], status inequality [3], lack of clarity around participants’ roles [3] and unequal participation [14]. Emphasis on single disciplinary perspectives is most likely when one or two members only dominate the discussion, disallowing collaboration from the rest of the Multidisciplinary Group [21]. It is important that clear goals and objectives [22] are sufficiently explained as this may have undermined the collaborative potential of the Multidisciplinary Group.

This study makes several assumptions. Firstly, the importance of a subject within a conversation is indicated by the amount of time the participants spend on it. Secondly, the professional groups and individuals involved were poorly integrated at the outset. Thirdly, by meeting on a regular basis to discuss individual clinical cases, it is this process and not some other parallel or coincident event that explains any perceived or actual improvement in integration. Finally, integrating within a Multidisciplinary Group can translate to meaningful and enduring integration in practice outside of the Multidisciplinary Group meetings.

Caution should be exercised before extrapolating the communication patterns of individuals to their professional category [21]. Although consultants appeared to exercise disproportionate participation within the Multidisciplinary Group, this needs to be interpreted relative to context and expectations [23]. Participation might not mean power, and even if it does Multidisciplinary Group participants might have little expectation or desire for communication to be any different.

We elected to identify patterns in the process of communication, rather than to describe narratives, power relationships, themes or outcomes. We used these patterns to examine evolutionary changes in the communication between the professional groups, with respect to integration and the involvement of the participants, supporting tentative conclusions about the functioning of Multidisciplinary Groups with respect to integration of organisations within a localised health economy. Also, we have tested the internal reliability of our coding scheme and found it acceptable. Quantifying qualitative data in this way raises philosophical and practical questions but employed pragmatically is useful to identify patterns in processes. We recognise the key advantage of our methodology is that evolutionary changes in the integrative intensity of the group might be evidenced over time by the regression line trending vertically. Comparisons between Multidisciplinary Groups can also be made in a visual and accessible way. Further research is needed to establish the regression line gradient that indicates whether integration is occurring during the meeting. Validating this technique against other measures of integration poses challenges because there is no gold standard measure of integration. However, this method allows researchers to critically assess whether the failure to integrate is a result of the qualitative characteristics of the discussion or the translation of that discussion into agreed actions. Based on our data, the assertion that Multidisciplinary Groups achieve virtual integration requires further substantiation and research. The method that we have extended here may be a useful tool moving forward.

## Conclusion

In the context of the integrated care agenda, we believe that this is the first attempt to empirically measure integration between participants in a Multidisciplinary Group [24]. The coding scheme and the analytical strategy that we have employed may be a useful tool for participants, managers and commissioners to measure how well Multidisciplinary Groups are delivering on their objectives and to identify barriers and challenges in doing so.

## Reviewers

**Heather Boon**, Interim Dean, Leslie Dan Faculty of Pharmacy, University of Toronto, Canada

**Gina Browne**, Professor, Health and Social Service Utilization Research Unit, Canada

**Nuria Toro**, Senior Researcher, Basque Foundation for Healthcare Innovation and Research, Spain

## Figures and Tables

**Table 1.**
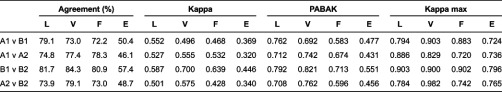
Agreement, Kappa, Prevalence and Bias-Adjusted Kappa and Kappa max

L, Level; V, Valence; F, Focus; E, trivalent Event Code.
